# Correlation analysis reveals the emergence of coherence in the gene expression dynamics following system perturbation

**DOI:** 10.1186/1471-2105-8-S1-S16

**Published:** 2007-03-08

**Authors:** Nicola Neretti, Daniel Remondini, Marc Tatar, John M Sedivy, Michela Pierini, Dawn Mazzatti, Jonathan Powell, Claudio Franceschi, Gastrone C Castellani

**Affiliations:** 1 Institute for Brain and Neural Systems, Brown University, Providence RI, USA; 2 Centro Interdipartimentale "L. Galvani", Università di Bologna, Bologna, Italy; 3 Department of Ecology and Evolutionary Biology, Brown University, Providence RI, USA; 4 Department of Molecular Biology, Cell Biology and Biochemistry, Brown University, Providence RI, USA; 5 Unilever Corporate Research Center, Colworth, UK; 6 I.N.R.C.A., Department of Gerontological Sciences, via Birarelli 8, 60121 Ancona, Italy; 7 DIMORFIPA, Università di Bologna, Bologna, Italy

## Abstract

Time course gene expression experiments are a popular means to infer co-expression. Many methods have been proposed to cluster genes or to build networks based on similarity measures of their expression dynamics. In this paper we apply a correlation based approach to network reconstruction to three datasets of time series gene expression following system perturbation: 1) Conditional, Tamoxifen dependent, activation of the cMyc proto-oncogene in rat fibroblast; 2) Genomic response to nutrition changes in *D. melanogaster*; 3) Patterns of gene activity as a consequence of ageing occurring over a life-span time series (25y–90y) sampled from T-cells of human donors.

We show that the three datasets undergo similar transitions from an "uncorrelated" regime to a positively or negatively correlated one that is symptomatic of a shift from a "ground" or "basal" state to a "polarized" state.

In addition, we show that a similar transition is conserved at the pathway level, and that this information can be used for the construction of "meta-networks" where it is possible to assess new relations among functionally distant sets of molecular functions.

## Background

Time series of gene expression data from high throughput experiments have been used to infer networks of co-expressed genes. By following the changes in expression at the genomic level, it is possible to identify groups of genes with a similar expression pattern. Most of the techniques currently used in functional genomics have been adapted from machine learning and statistical inference. Some of them generate networks of genes; others simply generate clusters of genes. Examples of the latter are algorithms based on Self-Organizing Maps (SOM) [1-4], Phylogenic-Type Trees [5-10], agglomerative clustering and partitional clustering. For network determination, techniques have been developed based on differential equations [11], Bayesian networks [12], hybrid petri networks [13], Boolean regulatory networks [14,15].

Relevance networks [16,17] are a popular method for the analysis of time series of expression levels. The basic idea is to construct a network of similarity of the genes' expression patterns. Several similarity measures have been utilized, such as correlation and mutual information. This technique can then represent multiple connections between genes, and capture negative as well as positive correlations. Once the matrix containing the similarity measure for all pairs of genes has been computed, a threshold is used to define the links in the network. Network validation can be obtained by permutation testing, i.e. by randomly shuffling the time points independently for each gene.

A similar approach has been applied to metabolic networks [18,19]. The authors computed metabolite correlations to infer changes in regulation using samples from different physiological states. On the other hand, we focused on the analysis of time series of expression levels for genes that were selected for differential expression between treatment and control.

An alternative approach is offered by Graphical Gaussian Models (GGM) that use partial correlation as a measure of independence between two genes. Partial correlations are related to the inverse of the correlation matrix, and in GGMs missing edges indicate conditional independence. One of the biggest problems with GGMs is that the number of genes is usually much larger than the number of samples (e.g. time points), such that the correlation matrix is usually singular and cannot be inverted. Different approaches have been proposed to circumvent this problem: restrict the number of genes analyzed to less than the number of samples [20-22]; use partial correlation coefficients of limited order [23-25]; approach the matrix inversion as an ill-posed inverse problem through regularization methods (usually via empirical Bayes, such as variance reduction) [26,27].

Although co-expression is not a direct indication of co-regulation, it is a very useful tool that can be used to interpret the effect of a perturbation in eliciting different phenotypes when combined with an ontology analysis. Here, we use a correlation based method to generate co-expression networks on three different datasets and we characterize the change in its structural properties induced by the system's perturbation. We then use a correlation-based analysis to infer how the perturbation affects the system at the level of metabolic and signalling pathways.

## Results

We considered three datasets of time course gene expression arrays: 1) Conditional, Tamoxifen dependent, activation of the cMyc proto-oncogene in rat fibroblast; 2) Genomic response to nutrition changes in *D. melanogaster*; 3) Patterns of gene activity as a consequence of ageing occurring over a life-span time series (25y–90y) sampled from T-cells of human donors.

### Gene selection

Due to the heterogeneous nature and properties of the datasets, we selected the significant genes according to different criteria.

For the cMyc dataset we applied a two way ANOVA (time and treatment as variability factors) to identify genes whose expression pattern was significantly affected by cMyc activation. With this method we identified a set of 1,191 significant genes out of a total of 8,799 [28].

The *D. melanogaster *diet arrays provide a higher resolution dataset and genes were selected via GeneTrace, which looks for change points in the time series of the expression ratios between the two cohorts. GeneTrace identified 3,519 genes with significant change point. These results showed that physiological response to nutrient uptake involves a rapid change in transcriptional profile at a global scale, and that most of the changes are small (81% of the 3,519 ratios smaller than 1.5 fold).

For the human aging dataset we applied one way ANOVA (with donors' age as variability factor) with a *P *value < 0.01 that resulted in a set of 768 probesets selected out of a total of 14,688 probesets.

### Correlation network analysis

**Figure 1:**
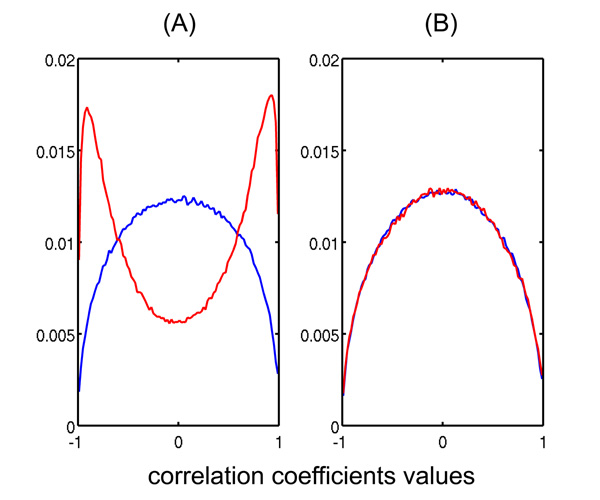
**Histogram of correlation coefficients of the gene expression time series between genes for the cMyc dataset**. The red line refers to the perturbed case, whereas the blue to the unperturbed one. (A) The perturbation induces a bimodal distribution: genes tend to be either strongly correlated or anti-correlated, differing significantly from unperturbed case. (B) Correlation coefficient histograms obtained after time reshuffling of the same genes do not show any significant difference.

When cMyc is activated by Tamoxifen, the activity profile of the probesets clearly changes into a strongly correlated regime. These findings are reflected in the histograms of the correlation coefficients for the *N-*control and *T-*treatment data sets (Figure 1) and in the main parameters of the connectivity distributions obtained from the corresponding adjacency matrices (Table 1). The adjacency matrix characterizing the network was obtained by considering only the correlation coefficients whose absolute value exceeded a threshold fixed between 0.95 and 0.99 (we remark that the lower threshold value was higher than the value requested for a P < 0.05 statistical significance of the correlation coefficients). The results shown in this paper were obtained for a threshold equal to 0.98, but similar results held for the [0.95–0.99] interval. These coefficients were set equal to 1, producing a symmetric adjacency matrix *a*_*lr*_. For each gene connectivity degree *k *was defined as the total number of genes it was connected to, i.e.

k(l)=∑r≠lalr
 MathType@MTEF@5@5@+=feaafiart1ev1aaatCvAUfKttLearuWrP9MDH5MBPbIqV92AaeXatLxBI9gBaebbnrfifHhDYfgasaacH8akY=wiFfYdH8Gipec8Eeeu0xXdbba9frFj0=OqFfea0dXdd9vqai=hGuQ8kuc9pgc9s8qqaq=dirpe0xb9q8qiLsFr0=vr0=vr0dc8meaabaqaciaacaGaaeqabaqabeGadaaakeaacqWGRbWAcqGGOaakcqWGSbaBcqGGPaqkcqGH9aqpdaaeqbqaaiabdggaHnaaBaaaleaacqWGSbaBcqWGYbGCaeqaaaqaaiabdkhaYjabgcMi5kabdYgaSbqab0GaeyyeIuoaaaa@3D16@

For the *T*-treatment dataset the number of coefficients close to +1 or -1 increases significantly. This finding indicates that many of the 1,191 genes, whose expression levels over time were affected by tamoxifen stimulation, became either strongly correlated or anti-correlated.

**Figure 2:**
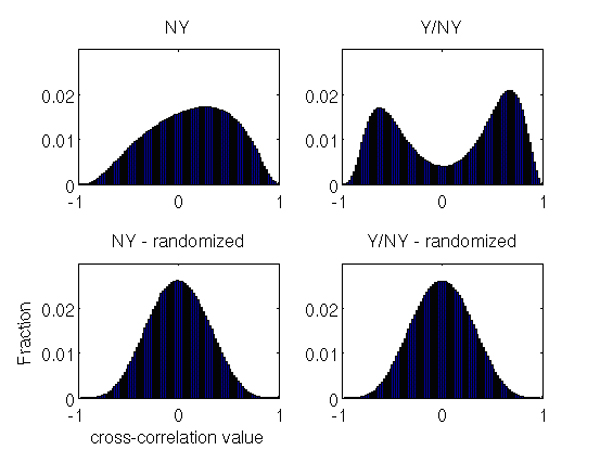
**Histogram of the correlation coefficients of the gene expression time series between all the genes selected with the change point analysis in the *D. melanogaster *dataset**. In the NY-controls (top left) the histogram resembles a Gaussian distribution slightly skewed towards positive correlation values. When considering the expression ratio Y-treatment over NY-control (top right) the distribution becomes bimodal and genes tend to be either strongly correlated or anti-correlated. These results have been validated by reshuffling the time points independently for each gene. In both cohorts this leads to a Gaussian distribution (bottom row).

In Figure 2 we show the histogram of the correlation coefficients between all the genes selected with the change point analysis in the *D. melanogaster *dataset. In the NY-controls (top left) the histogram resembles a Gaussian distribution slightly skewed towards positive correlation values. When considering the expression ratio Y-treatment over NY-control (top right) the distribution becomes bimodal and genes tend to be either strongly correlated or anti-correlated.

**Figure 3:**
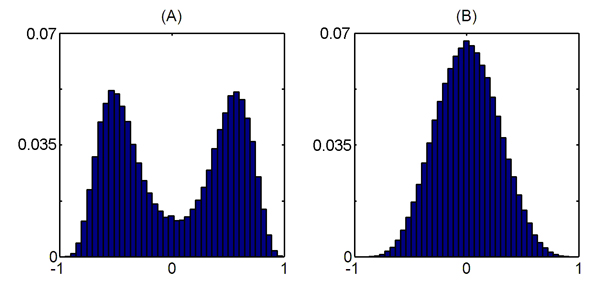
**Histogram of correlation coefficients of the gene expression time series between genes for the aging dataset**. The picture on the left (A) shows the histogram of the correlation coefficients for the set of 768 probesets selected with one-way ANOVA, P value < 0.01. The picture on the right (B) is the histogram of the correlation coefficients for a set of 768 probesets randomly sampled from the whole dataset of 14688 probesets. A single-gene time reshuffling applied onto each dataset produces a Gaussian distribution (data not shown).

We used time reshuffling to test the time sequence dependence of the results. By randomly shuffling the time series for each gene separately, time relationships between expression levels are broken, but the mean and standard deviation for each gene are unaltered. Properties of the gene network that truly depend on the expression level dynamics should be significantly affected by a random shuffling in time. The bottom row of Figure 2 shows the result for the *D. melanogaster *data. In both cohorts this leads to a Gaussian distribution for the values of the correlation coefficients (Fig. 2 bottom row). Analogous results have been obtained for the other two datasets (Fig. 1 and Fig. 3).

The role of noise was taken in to account by analyzing the correlation coefficient distribution obtained from a dataset of randomly generated vectors of the same size than the experimental datasets (e.g. for the c-Myc dataset: a 5 × 1191 array of values sampled from the Standardized Gaussian Distribution [29]). The resulting distribution strictly resambles that obtained with the no Tamoxifen dataset.

**Figure 4:**
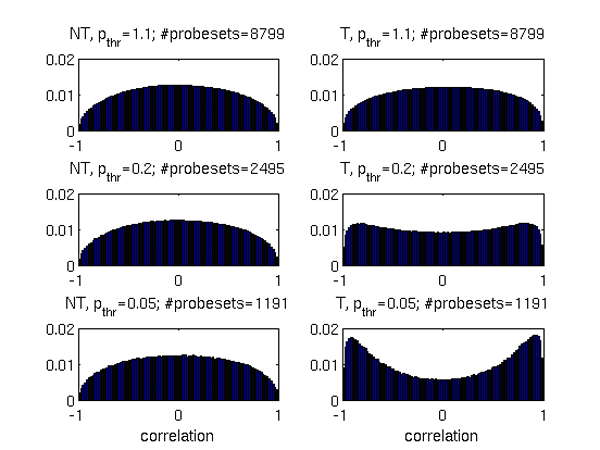
**Transition from unimodal to bimodal behavior for the cMyc data**. The different panels show the histogram of the correlation coefficients between the expression values over time from the probesets in the dataset N (left column) and T (right column) for decreasing cutoff *P *values (*P*_*thr*_). The top row includes all the probesets used in the analysis (*P*_*thr *_> 1); the central row corresponds to an intermediate threshold (*P*_*thr *_= 0.2); the bottom row corresponds to the lowest threshold (*P*_*thr *_= 0.05).

It is possible to identify a subnetwork of strongly correlated/anticorrelated genes by selecting the probesets based on the the treatment factor's strength in the ANOVA analysis. This selection process can be characterized as a transition from a unimodal (most genes are uncorrelated) to a bi-modal behavior (genes are either correlated or anticorrelated). Figure 4 shows how this process occurs by decreasing the cutoff *P *value (*P*_*thr*_) used to select significant genes in the cMyc dataset. The different panels show the histogram of the correlation coefficients between the expression values over time from the probesets in the dataset N (left column) and T (right column) for decreasing *P*_*thr*_. The top row includes all the probesets used in the analysis (*P*_*thr *_> 1); the central row corresponds to an intermediate threshold (*P*_*thr *_= 0.2); the bottom row corresponds to the lowest threshold (*P*_*thr *_= 0.05). Notice that the transition from unimodal to bimodal is only present in the T dataset, while the N dataset in not affected even when the cutoff *P *value is very small. Analogous results have been obtained for the other two datasets.

### Pathway analysis

We identified the pathways whose genes' expression was significantly affected by the system perturbation. We performed this analysis on the *D. melanogaster *diet dataset and on the *cMyc *dataset which share the common feature of being generated through a direct perturbation of the system. The problem in this analysis is that different pathways in the KEGG [30] database contain different numbers of genes, and that not all of them had been measured due to limitations in the microarrays utilized. For this reason we computed the 95% confidence limits for the population proportions (*p*) of affected genes in each pathway (see Methods). We found that 45 pathways out of 110 included at least 20% of genes whose expression pattern changed significantly upon re-feeding (see Table 2). For the cMyc dataset, a smaller proportion of genes had been measured in each pathway. Hence, we used a lower threshold and found that 51 pathways out of 135 had more than 5% of the measured genes with significant changes (Table 3).

**Figure 5:**
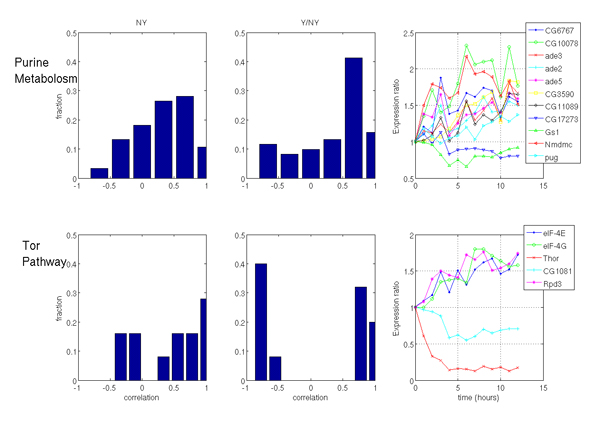
**Histogram of the correlation coefficients between all the genes selected within the purine synthesis pathway (top row) and Tor pathway (bottom row)**. The expression ratios for the single genes are shown in the rightmost panels. The difference in the correlation distributions between Y-treatment and NY-control is qualitatively the same as the one observed in the entire pool of genes selected with the change point analysis (Figure 2).

**Figure 6:**
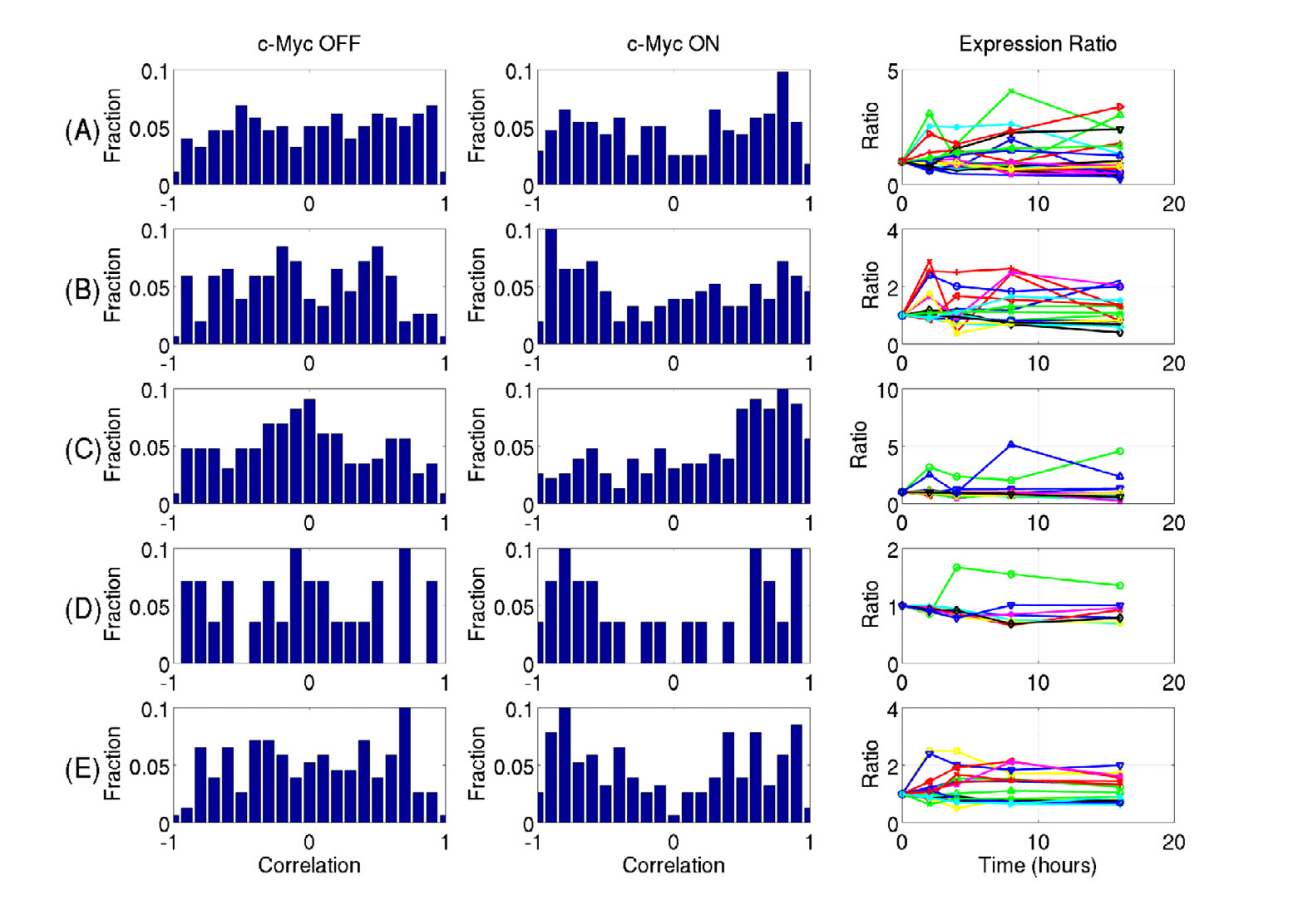
**Histogram of the correlation coefficients between all the genes for selected pathways**. (A) MAPK signaling, (B) calcium signaling, (C) focal adhesion, (D) gap junction, (E) insulin/IGF signaling. The expression ratios for the single genes in each pathway are shown in the rightmost panels. The difference in the correlation distributions between c-Myc-ON-treatment (central panel) and c-Myc-OFF-control (left panel) is qualitatively the same as the one observed in the entire pool of genes selected with the ANOVA analysis (Fig. 1).

When we analyzed these pathways with the correlation method described earlier, we found that the difference in the correlation distributions between treated and untreated cases is qualitatively the same as the one observed in the entire pool of genes selected with the change point analysis. Figure 5 shows this effect, as well as the time course of the expression ratios for the Purine metabolism pathway and the Target of Rapamycin (TOR) pathway (notice that TOR does not compare in the Table 2, since it is not part of the KEGG database and was instead manually annotated from the literature). The same effect was observed in the cMyc dataset, as exemplified in Figure 6 for five representative pathways: (A) MAPK signaling, (B) calcium signaling, (C) focal adhesion, (D) gap junction, (E) insulin/IGF signaling.

## Discussion

In all three data sets different and specific genes are affected by the perturbations but give rise to a common correlation profile (Figures 1, 2, 3). This suggests that, despite their specificity, the global changes in gene expression follow a pattern that is largely independent from the data sets. This results could be explained by assuming that temporal changes in gene expression in biological complex systems is scale-independent and characterized by changes in an initial triggering core of genes (specific to the perturbation and the cell type) followed by a propagation to other genes.

In particular, in the cMyc dataset we found that the correlation method identifies a list of genes containing many of the genes found by O'Connell et al. [31], as well as many genes that, to our knowledge, have not been identified before. This indicates the possibility that the cMyc regulatory network may be much larger than currently described. To rule out the presence of undetectable correlations between genes in the unperturbed state (i.e. when cMyc is not active), we performed a one-way analysis of variance on the unperturbed dataset to detect genes whose expression level changes significantly over time. Less than 3% of the genes was selected for differential expression over time, compared to more than 10% for the perturbed dataset (P < 0.05).

For the *D. melanogaster *dataset our analysis showed that refeeding induces a change in the expression patterns of thousands of genes. Since this analysis was performed at saturation, we estimate there are few false positives and few undetected changes. The analysis of the correlation between expression patterns reveals that many genes respond to re-feeding in a coordinated manner. In fact, upon re-feeding, the activity profile of the genes clearly changes to a stronglycorrelated/anticorrelated regime.

**Figure 7:**
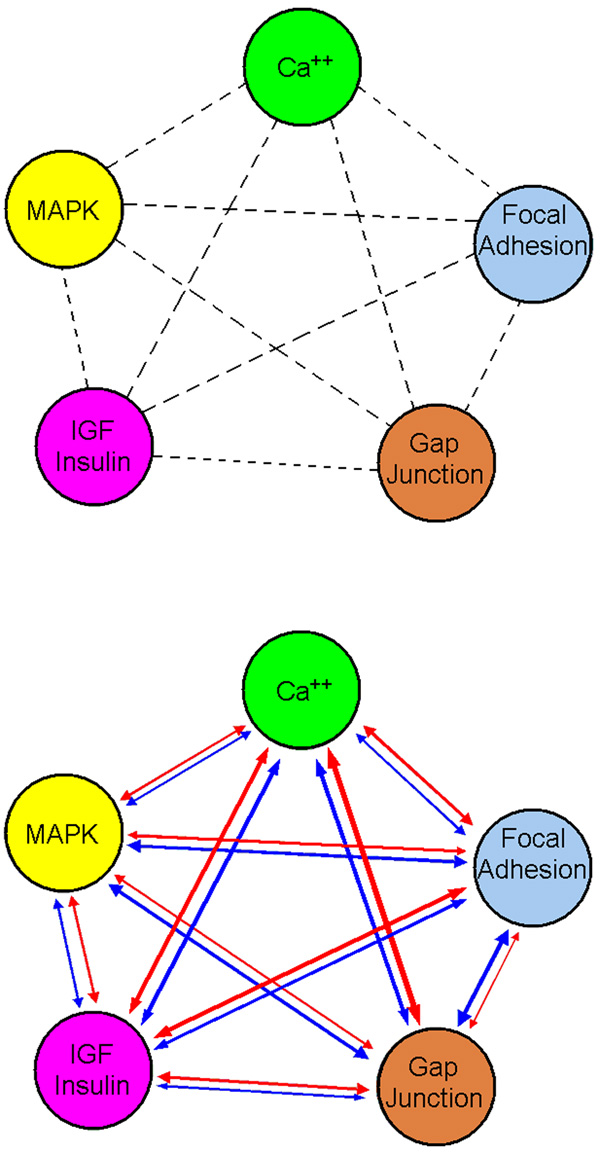
**Network between the 5 selected pathways of Fig in the case of C-MYC off. All these pathways show weak co-expression (dotted lines)**. (A) When cMyc- is on, pathways show positive and negative correlations (B). The red and blue arrows denote positive and negative co-regulation, respectively. The thickness of the arrows is proportional to the magnitude, or absolute value, of the co-expression.

An extension of this method, based on the cross-correlation between genes belonging to different pathways allowed us to build a network of relationships between pathways. In Figure 7 we show changes of a five-nodes network of selected genes when cMyc is activated. The five pathways, MAPK signaling, calcium signaling (CS), focal adhesion (FA), gap junction (GJ), insulin/IGF signaling (INS/IGF), were among those selected for the large proportion of significant genes (Table 3). The unperturbed state is characterized by a dominance of random correlations between pathways whereas after the perturbation we observed the emergence of positive and negative correlation regimes (Figure 6).

The MAPK showed a marked increase in negative correlation with CS and INS/IGF, a doubling in positive correlation with FA and GJ. CS increased the number of negatively correlated genes with FA and doubled the number of both positively and negatively correlated genes with GJ. However there is a similar augment of positive and negative correlation between CS and INS/IGF. FA shows a very large increase in correlation with GJ and a large increase in the anticorrelation with INS/IGF. On the contrary, GJ shows a very large increase in the anticorrelation with INS/IGF accompanied to a marked increase in correlation. It appears that MAPK, FA and GJ become more correlated between them and more anticorrelated with CS and INS/IGF.

Overall, our analysis revealed the emergence of positive links between MAPK, FA and GJ in following cMyc activation, an interesting insight given that the relation between electrical cell properties and the signaling system is, without a doubt, an important step for tumor establishment and progression. The increase in the anticorrelation of MAPK, FA and GJ with CS and INS/IGF may be interpreted as a new kind of control by CS and INS/IGF of the other three pathways.

## Conclusion

Three different systems sharing the time series experimental design after a perturbation (cMyc conditional activation, refeeding after caloric restriction and effect of time in young and old donors) have been examined.

At the genomic scale, we showed that in all three datasets the correlation regime between genes selected by statistical significance analysis follow a similar behavior, which is compatible with the emergence of coherence in the gene expression dynamics following cell perturbation. We also showed that this behavior is conserved at the pathways scale for pathways that have been significantly affected by the perturbation.

In particular, our analysis revealed the emergence of links between a core set of pathways in the cMyc dataset which may play an important role for the comprehension of the early phenotypical changes following cMyc activation.

This method was successful in identifying changes in gene expression profiles related to the acute response to a perturbation both in model systems and in humans as well as in revealing the centrality and importance of selected pathways by its multiscale generalization.

## Methods

### Microarray datasets

#### 1) cMyc dataset

Two gene expression data sets were extracted from the set of microarray experiments based on genetically engineered rat cell lines [31]. The first data set (*N *data set) contains the gene expression data for the c *-myc*^-/- ^MycER cell line treated with vehicle (ethanol) only. The second data set (*T *data set) contains the gene expression data collected after the addition of tamoxifen. Binding of tamoxifen to the estrogen receptor domain elicits a conformational change that allows the fusion protein to migrate to the nucleus and act as a transcription factor. Samples were harvested at five time points after the addition of tamoxifen to the culture medium: 1, 2, 4, 8, and 16 h. The entire experiment was repeated on three separate occasions, providing three independent measurements for each gene and each time point. Expression profiling was done by using the Affymetrix platform and U34A Gene Chips.

#### 2) *D. melanogaster *diet dataset

Newly enclosed virgin females were maintained in yeast-free media until 4 days old and then transferred either to media with yeast (Y-treatment) or to control media without yeast (NY-control). Samples were collected every hour for the following 12 hours. Four additional samples were collected prior to refeeding and arbitrarily designated -4, -3, -2, and -1. Synchronization of the physiological state was obtained by imposing diet restriction in third instars. Affymetric gene chips were used to measure mRNA abundance at each hour for both Y-treatment and NY-controls. A time-ordered sequence of expression ratios was then computed for each gene in the array [32].

#### 3) Human aging dataset

T-cells were extracted from peripheral blood of 20 healthy male human donors. Donors were age-stratified into 4 groups of 5 subjects each: 25–35 years old, 40–50 years old, 55–65 years old and 70–80 years old respectively. Custom-made microarrays (Unilever Labs, Colworth UK) with about 19000 human probes were performed in duplicate (dye-swap) for each subject.

### Probeset selection

#### 1) cMyc dataset

A full factorial ANOVA was applied to each of the 8,799 probesets to identify those that significantly changed in their expression level in time between the two conditions (data set *N *versus data set *T*). Probesets with a *P *value corresponding to the change-in-treatment factor < 0.05 were considered to be significantly affected by the treatment (i.e., activation of Myc by tamoxifen). A total of 1,191 genes were selected using this criterion.

#### 2) *D. melanogaster *diet dataset

When considering high resolution time series, change-point analysis [33] is a powerful tool for detecting subtle changes; it is robust to outliers and can control the overall error rate. The Tatar group has extended this technique to statistically detect changes for time-series microarray data (GeneTrace [32]). For each gene's time-series, the algorithm returns an estimated change-point (CUSUM estimator) and the two-tailed probability of this statistic from a sample of 10,000 random permutations across all of the ordered expression ratios. GeneTrace identified 3,519 genes with significant change point (significance threshold = 0.005, expected false positive inferences 70 out of 14,000).

#### 3) Human aging dataset

One-way ANOVA was applied to each of the 14,688 probesets to identify those that significantly change their expression level in time. With a *P *value of 0.01, 768 genes were selected for further analysis.

### Correlation analysis

The similarity measure for the expression dynamics of two genes within the same data set is given by the correlation between the two expression-level time series. For a given data set, if *x*_*lj *_is the expression level of a gene with label *l *at time *j*, then the similarity between two genes with labels *l *and *r*, respectively, is given by:

clr=∑j(xlj−μl)(xrj−μr)σlσr,
 MathType@MTEF@5@5@+=feaafiart1ev1aaatCvAUfKttLearuWrP9MDH5MBPbIqV92AaeXatLxBI9gBaebbnrfifHhDYfgasaacH8akY=wiFfYdH8Gipec8Eeeu0xXdbba9frFj0=OqFfea0dXdd9vqai=hGuQ8kuc9pgc9s8qqaq=dirpe0xb9q8qiLsFr0=vr0=vr0dc8meaabaqaciaacaGaaeqabaqabeGadaaakeaacqWGJbWydaWgaaWcbaGaemiBaWMaemOCaihabeaakiabg2da9maalaaabaWaaabeaeaadaqadaqaaiabdIha4naaBaaaleaacqWGSbaBcqWGQbGAaeqaaOGaeyOeI0ccciGae8hVd02aaSbaaSqaaiabdYgaSbqabaaakiaawIcacaGLPaaadaqadaqaaiabdIha4naaBaaaleaacqWGYbGCcqWGQbGAaeqaaOGaeyOeI0Iae8hVd02aaSbaaSqaaiabdkhaYbqabaaakiaawIcacaGLPaaaaSqaaiabdQgaQbqab0GaeyyeIuoaaOqaaiab=n8aZnaaBaaaleaacqWGSbaBaeqaaOGae83Wdm3aaSbaaSqaaiabdkhaYbqabaaaaOGaeiilaWcaaa@516F@

where *μ*_l _and *μ*_r _are the averages in time of the expression levels for the two genes, and *σ*_l _and *σ*_r _are their standard deviations.

Time reshuffling was used to test the time sequence dependence of the results obtained by the two techniques. By randomly shuffling the time series for each gene separately, time relationships between expression levels are broken, but the mean and standard deviation for each gene are unaltered. Properties of the gene network that truly depend on the expression level dynamics should be significantly affected by a random shuffling in time.

### Pathway analysis

We ranked pathways based on the percentage of genes selected by the significance analysis in each one of them. To account for the different number of measured genes in different pathways, we computed the 95% confidence limits for the population proportions (*p*) of significant genes in each pathway. Using a relationship between the *F *distribution and the binomial distribution [34], the confidence interval can be computed for the binomial parameter *p *[35-37]. The lower confidence limit *L*_1 _and the upper confidence limit *L*_2 _become:

L1=XX+(n−X+1)Fα(2),ν1,ν2,ν1=2(n−X+1),ν2=2XL2=(X+1)Fα(2),ν′1,ν′2n−X+(X+1)Fα(2),ν′1,ν′2,ν′1=2(X+1),ν′2=2(n−X)
 MathType@MTEF@5@5@+=feaafiart1ev1aaatCvAUfKttLearuWrP9MDH5MBPbIqV92AaeXatLxBI9gBaebbnrfifHhDYfgasaacH8akY=wiFfYdH8Gipec8Eeeu0xXdbba9frFj0=OqFfea0dXdd9vqai=hGuQ8kuc9pgc9s8qqaq=dirpe0xb9q8qiLsFr0=vr0=vr0dc8meaabaqaciaacaGaaeqabaqabeGadaaakeaafaqabeGacaaabaGaemitaW0aaSbaaSqaaiabigdaXaqabaGccqGH9aqpdaWcaaqaaiabdIfaybqaaiabdIfayjabgUcaRiabcIcaOiabd6gaUjabgkHiTiabdIfayjabgUcaRiabigdaXiabcMcaPiabdAeagnaaBaaaleaaiiGacqWFXoqycqGGOaakcqaIYaGmcqGGPaqkcqGGSaalcqWF9oGBdaWgaaadbaGaeGymaedabeaaliabcYcaSiab=17aUnaaBaaameaacqaIYaGmaeqaaaWcbeaaaaGccqGGSaalaeaacqWF9oGBdaWgaaWcbaGaeGymaedabeaakiabg2da9iabikdaYiabcIcaOiabd6gaUjabgkHiTiabdIfayjabgUcaRiabigdaXiabcMcaPiabcYcaSiab=17aUnaaBaaaleaacqaIYaGmaeqaaOGaeyypa0JaeGOmaiJaemiwaGfabaGaemitaW0aaSbaaSqaaiabikdaYaqabaGccqGH9aqpdaWcaaqaaiabcIcaOiabdIfayjabgUcaRiabigdaXiabcMcaPiabdAeagnaaBaaaleaacqWFXoqycqGGOaakcqaIYaGmcqGGPaqkcqGGSaalcuWF9oGBgaqbamaaBaaameaacqaIXaqmaeqaaSGaeiilaWIaf8xVd4MbauaadaWgaaadbaGaeGOmaidabeaaaSqabaaakeaacqWGUbGBcqGHsislcqWGybawcqGHRaWkcqGGOaakcqWGybawcqGHRaWkcqaIXaqmcqGGPaqkcqWGgbGrdaWgaaWcbaGae8xSdeMaeiikaGIaeGOmaiJaeiykaKIaeiilaWIaf8xVd4MbauaadaWgaaadbaGaeGymaedabeaaliabcYcaSiqb=17aUzaafaWaaSbaaWqaaiabikdaYaqabaaaleqaaaaakiabcYcaSaqaaiqb=17aUzaafaWaaSbaaSqaaiabigdaXaqabaGccqGH9aqpcqaIYaGmcqGGOaakcqWGybawcqGHRaWkcqaIXaqmcqGGPaqkcqGGSaalcuWF9oGBgaqbamaaBaaaleaacqaIYaGmaeqaaOGaeyypa0JaeGOmaiJaeiikaGIaemOBa4MaeyOeI0IaemiwaGLaeiykaKcaaaaa@9C23@

where n is the total number of genes measured in a pathway, and X is the number of significant genes in that pathway.

We selected a 20% threshold for the Drosophila array and 5% threshold for the rat array. In fact, the Drosophila array contained 14,688 probesets corresponding to more than 90% of the fly's genome. Hence the analysis was done at saturation. The rat array used in the cMyc experiments contained 8,799 probesets corresponding to roughly 1/3 of the rat's genome. Requiring a 20% change in the cMyc experiment would result in a very small list of pathways. However, it is well known that cMyc affects the expression of a large pool of genes (directly or indirectly). Our interpretation is that the same cMyc experiment done at saturation would reflect this at the pathway level as well, i.e. many pathways would more than 20% of the genes with a significant change in expression profile over time.

Links between selected pathways were generated by computing the correlation of the gene expression time series between genes in different pathways and generating a block-correlation matrix with entries CPiPjX
 MathType@MTEF@5@5@+=feaafiart1ev1aaatCvAUfKttLearuWrP9MDH5MBPbIqV92AaeXatLxBI9gBaebbnrfifHhDYfgasaacH8akY=wiFfYdH8Gipec8Eeeu0xXdbba9frFj0=OqFfea0dXdd9vqai=hGuQ8kuc9pgc9s8qqaq=dirpe0xb9q8qiLsFr0=vr0=vr0dc8meaabaqaciaacaGaaeqabaqabeGadaaakeaaieqacqWFdbWqdaqhaaWcbaacbmGae4huaa1aaSbaaWqaaiab+LgaPbqabaWccqGFqbaudaWgaaadbaGae4NAaOgabeaaaSqaaiab+Hfaybaaaaa@3494@ that are respectively the correlation of the gene expression time series between the genes in pathways *P*_*i *_and *P*_*j*_.

[CP1P1XCP1P2X⋯CP1PmXCP2P1XCP2P2X⋯CP2PmX⋮⋮⋱⋮CPmP1XCPmP2X⋯CPmPmX]
 MathType@MTEF@5@5@+=feaafiart1ev1aaatCvAUfKttLearuWrP9MDH5MBPbIqV92AaeXatLxBI9gBaebbnrfifHhDYfgasaacH8akY=wiFfYdH8Gipec8Eeeu0xXdbba9frFj0=OqFfea0dXdd9vqai=hGuQ8kuc9pgc9s8qqaq=dirpe0xb9q8qiLsFr0=vr0=vr0dc8meaabaqaciaacaGaaeqabaqabeGadaaakeaadaWadaqaauaabeqaeqaaaaaabaacbeGae83qam0aa0baaSqaaGqadiab+bfaqnaaBaaameaacqaIXaqmaeqaaSGae4huaa1aaSbaaWqaaiabigdaXaqabaaaleaacqGFybawaaaakeaacqWFdbWqdaqhaaWcbaGae4huaa1aaSbaaWqaaiabigdaXaqabaWccqGFqbaudaWgaaadbaGaeGOmaidabeaaaSqaaiab+HfaybaaaOqaaiabl+Uimbqaaiab=neadnaaDaaaleaacqGFqbaudaWgaaadbaGaeGymaedabeaaliab+bfaqnaaBaaameaacqGFTbqBaeqaaaWcbaGae4hwaGfaaaGcbaGae83qam0aa0baaSqaaiab+bfaqnaaBaaameaacqaIYaGmaeqaaSGae4huaa1aaSbaaWqaaiabigdaXaqabaaaleaacqGFybawaaaakeaacqWFdbWqdaqhaaWcbaGae4huaa1aaSbaaWqaaiabikdaYaqabaWccqGFqbaudaWgaaadbaGaeGOmaidabeaaaSqaaiab+HfaybaaaOqaaiabl+Uimbqaaiab=neadnaaDaaaleaacqGFqbaudaWgaaadbaGaeGOmaidabeaaliab+bfaqnaaBaaameaacqGFTbqBaeqaaaWcbaGae4hwaGfaaaGcbaGaeSO7I0eabaGaeSO7I0eabaGaeSy8I8eabaGaeSO7I0eabaGae83qam0aa0baaSqaaiab+bfaqnaaBaaameaacqGFTbqBaeqaaSGae4huaa1aaSbaaWqaaiabigdaXaqabaaaleaacqGFybawaaaakeaacqWFdbWqdaqhaaWcbaGae4huaa1aaSbaaWqaaiab+1gaTbqabaWccqGFqbaudaWgaaadbaGaeGOmaidabeaaaSqaaiab+HfaybaaaOqaaiabl+Uimbqaaiab=neadnaaDaaaleaacqGFqbaudaWgaaadbaGae4xBa0gabeaaliab+bfaqnaaBaaameaacqGFTbqBaeqaaaWcbaGae4hwaGfaaaaaaOGaay5waiaaw2faaaaa@7E79@

## Authors' contributions

N. Neretti, D. Remondini. and G. C. Castellani developed the data analysis method, performed the analyses and wrote the paper; M. Tatar., J. M. Sedivy., M. Pierini., D. Mazzatti., J. Powell. designed and performed the experiments and the data collection; L. M. and C. F. supervised the analyses, suggested the biological implications, and wrote the paper.

## Figures and Tables

**Table 1:** Network parameters. Comparison of principal network parameters for the N and T datasets

**Network Parameters**	***N***	***T***
*k*_*min*_	0	0
*k*_*max*_	17	99
Mean, *k*	4.53	23.44
Standard deviation, *σ*(*k*)	2.61	23.97
Skewness *γ*(*k*)	0.89	1.16
Clustering coefficient *c*(*k*)	0.43	0.45

**Table 2:** List of pathways for *D. melanogaster *dataset. List of pathways (from the KEGG database) that include at least 20% of genes whose expression pattern has changed significantly upon re-feeding in the *D. melanogaster *dataset

**KEGG ID**	**Pathway Description**
path:dme00010	Glycolysis/Gluconeogenesis
path:dme00030	Pentose phosphate pathway
path:dme00051	Fructose and mannose metabolism
path:dme00052	Galactose metabolism
path:dme00053	Ascorbate and aldarate metabolism
path:dme00062	Fatty acid biosynthesis (path 2)
path:dme00071	Fatty acid metabolism
path:dme00120	Bile acid biosynthesis
path:dme00190	Oxidative phosphorylation
path:dme00230	Purine metabolism
path:dme00240	Pyrimidine metabolism
path:dme00251	Glutamate metabolism
path:dme00252	Alanine and aspartate metabolism
path:dme00260	Glycine, serine and threonine metabolism
path:dme00280	Valine, leucine and isoleucine degradation
path:dme00290	Valine, leucine and isoleucine biosynthesis
path:dme00310	Lysine degradation
path:dme00330	Arginine and proline metabolism
path:dme00340	Histidine metabolism
path:dme00350	Tyrosine metabolism
path:dme00361	Gamma-Hexachlorocyclohexane degradation
path:dme00380	Tryptophan metabolism
path:dme00410	Beta-Alanine metabolism
path:dme00440	Aminophosphonate metabolism
path:dme00500	Starch and sucrose metabolism
path:dme00562	Inositol phosphate metabolism
path:dme00564	Glycerophospholipid metabolism
path:dme00600	Glycosphingolipid metabolism
path:dme00620	Pyruvate metabolism
path:dme00624	1- and 2- Methylnaphthalene degradation
path:dme00625	Tetrachloroethene degradation
path:dme00632	Benzoate degradation via CoA ligation
path:dme00640	Propanoate metabolism
path:dme00650	Butanoate metabolism
path:dme00670	One carbon pool by folate
path:dme00740	Riboflavin metabolism
path:dme00790	Folate biosynthesis
path:dme00903	Limonene and pinene degradation
path:dme00920	Sulfur metabolism
path:dme00930	Caprolactam degradation
path:dme00970	Aminoacyl- tRNA biosynthesis
path:dme03020	RNA polymerase
path:dme03050	Proteasome
path:dme03060	Protein export
path:dme04070	Phosphatidylinositol signaling system

**Table 3:** List of pathways for *cMyc *dataset. List of pathways (from KEGG database) that include at least 5% of genes whose expression pattern has changed significantly upon cMyc activation

**KEGG ID**	**Pathway Description**	**KEGG ID**	**Pathway Description**
path:rno00020	Citrate cycle (TCA cycle)	path:rno00920	Sulfur metabolism
path:rno00051	Fructose and mannose metabolism	path:rno01510	Neurodegenerative Disorders
path:rno00052	Galactose metabolism	path:rno03010	Ribosome
path:rno00062	Fatty acid biosynthesis (path 2)	path:rno03020	RNA polymerase
path:rno00071	Fatty acid metabolism	path:rno03022	Basal transcription factors
path:rno00072	Synthesis and degradation of ketone bodies	path:rno03030	DNA polymerase
path:rno00230	Purine metabolism	path:rno04010	MAPK signaling pathway
path:rno00240	Pyrimidine metabolism	path:rno04020	Calcium signaling pathway
path:rno00280	Valine, leucine and isoleucine degradation	path:rno04070	Phosphatidylinositol signaling system
path:rno00310	Lysine degradation	path:rno04110	Cell cycle
path:rno00340	Histidine metabolism	path:rno04210	Apoptosis
path:rno00350	Tyrosine metabolism	path:rno04310	Wnt signaling pathway
path:rno00380	Tryptophan metabolism	path:rno04510	Focal adhesion
path:rno00440	Aminophosphonate metabolism	path:rno04520	Adherens junction
path:rno00480	Glutathione metabolism	path:rno04530	Tight junction
path:rno00510	N-Glycan biosynthesis	path:rno04540	Gap junction
path:rno00561	Glycerolipid metabolism	path:rno04620	Toll-like receptor signaling pathway
path:rno00564	Glycerophospholipid metabolism	path:rno04630	Jak-STAT signaling pathway
path:rno00590	Prostaglandin and leukotriene metabolism	path:rno04810	Regulation of actin cytoskeleton
path:rno00620	Pyruvate metabolism	path:rno04910	Insulin signaling pathway
path:rno00624	1- and 2-Methylnaphthalene degradation	path:rno05010	Alzheimer's disease
path:rno00630	Glyoxylate and dicarboxylate metabolism	path:rno05020	Parkinson's disease
path:rno00650	Butanoate metabolism	path:rno05030	Amyotrophic lateral sclerosis (ALS)
path:rno00720	Reductive carboxylate cycle (CO2 fixation)	path:rno05040	Huntington's disease
path:rno00790	Folate biosynthesis	path:rno05060	Prion disease
path:rno00903	Limonene and pinene degradation		
